# Comprehensive prediction of urolithiasis based on clinical factors, blood chemistry and urinalysis: UROLITHIASIS score

**DOI:** 10.1038/s41598-023-42208-9

**Published:** 2023-09-09

**Authors:** Hyo Joon Kim, Sang Hoon Oh

**Affiliations:** Department of Emergency Medicine, Seoul St. Mary’s Hospital, College of Medicine, The Catholic University of Korea, Seoul, 06509 Republic of Korea

**Keywords:** Ureter, Renal calculi, Urological manifestations

## Abstract

Comprehensive prediction of urolithiasis using available factors obtained in the emergency department may aid in patient-centered diagnostic imaging decisions. This retrospective study analyzed the clinical factors, blood chemistry and urine parameters of patients who underwent nonenhanced urinary computed tomography for suspected urolithiasis. A scoring system was developed from a logistic regression model and was tested using the area under the curve (AUC). The prevalence of urolithiasis and important possible causes in the three risk subgroups were determined. Finally, the scoring model was validated. In the derivation cohort (n = 673), 566 patients were diagnosed with urolithiasis. Age > 35 years, history of urolithiasis, pain duration < 8 h, nausea/vomiting, costovertebral angle tenderness, serum creatinine ≥ 0.92 mg/dL, erythrocytes ≥ 10/high power field, no leukocytes ≤  + , and any crystalluria were retained in the final multivariable model and became part of the score. This scoring model demonstrated good discrimination (AUC 0.808 [95% CI, 0.776–0.837]). In the validation cohort (n = 336), the performance was similar (AUC 0.803 [95% CI, 0.756–0.844]), surpassing that of the STONE score (AUC 0.654 [95% CI, 0.601–0.705], *P* < 0.001). This scoring model successfully stratified patients according to the probability of urolithiasis. Further validation in various settings is needed.

## Introduction

Urolithiasis is a commonly encountered problem in the emergency department (ED)^1^. The lifetime prevalence of urolithiasis is approximately 10–15% in developed countries^2^, and the relapse rate is approximately 75% within 20 years^3,4^. Moreover, the incidence of urolithiasis has been reported to be increasing globally^5,6^.

Since unenhanced computed tomography (CT) was first introduced for the diagnosis of urolithiasis in 1995^7^, it has become the gold-standard diagnostic tool for suspected ureteric colic^8,9^. The advantage of this diagnostic tool is that it is rapid and accurate, identifies the exact size and location of urolithiasis and has been successful at detecting other pathologic conditions^10^. Its main disadvantages are cost problems and radiation doses, which are coupled with the possible complications of an increased risk of cancer. Furthermore, recent literature shows that CT scans do not lead to improvements in patient outcomes^11,12^. Therefore, to optimize the diagnostic efficiency and determine patient-centered diagnostic strategies, with the recent increase in the use of point-of-care ultrasound (POCUS), several clinical prediction tools have been developed^13–16^. The best known and the most widely used is the STONE score^13^, which is the sum of integer points of five factors: sex (male), timing (duration of pain to presentation), origin, nausea, and erythrocytes in the urine dipstick test. Scores ranging from 0 to 5, 6–9 and 10–13 represent a low, moderate, and high probability of urolithiasis, respectively. The STONE score functions well and has been externally validated. However, contrary to the STONE score, which was derived from patients undergoing a nonenhanced CT (NECT) protocol for suspected urolithiasis, some validation studies have been conducted with various inclusions^16–18^. Furthermore, this scoring system does not include various test results available in the ED.

Therefore, we hypothesized that among consecutive patients undergoing a NECT protocol for suspected urolithiasis, a new comprehensive score based on clinical factors, blood chemistry and urinalysis (UROLITHIASIS score), would provide a more rational diagnostic strategy and aid in patient-centered diagnostic imaging decisions. The primary aim of this study was to develop and validate this comprehensive scoring model for patients who were highly suspected of having urolithiasis. The secondary aim was to validate and compare the published scoring system.

## Results

### Patients selection

**Figure 1:**
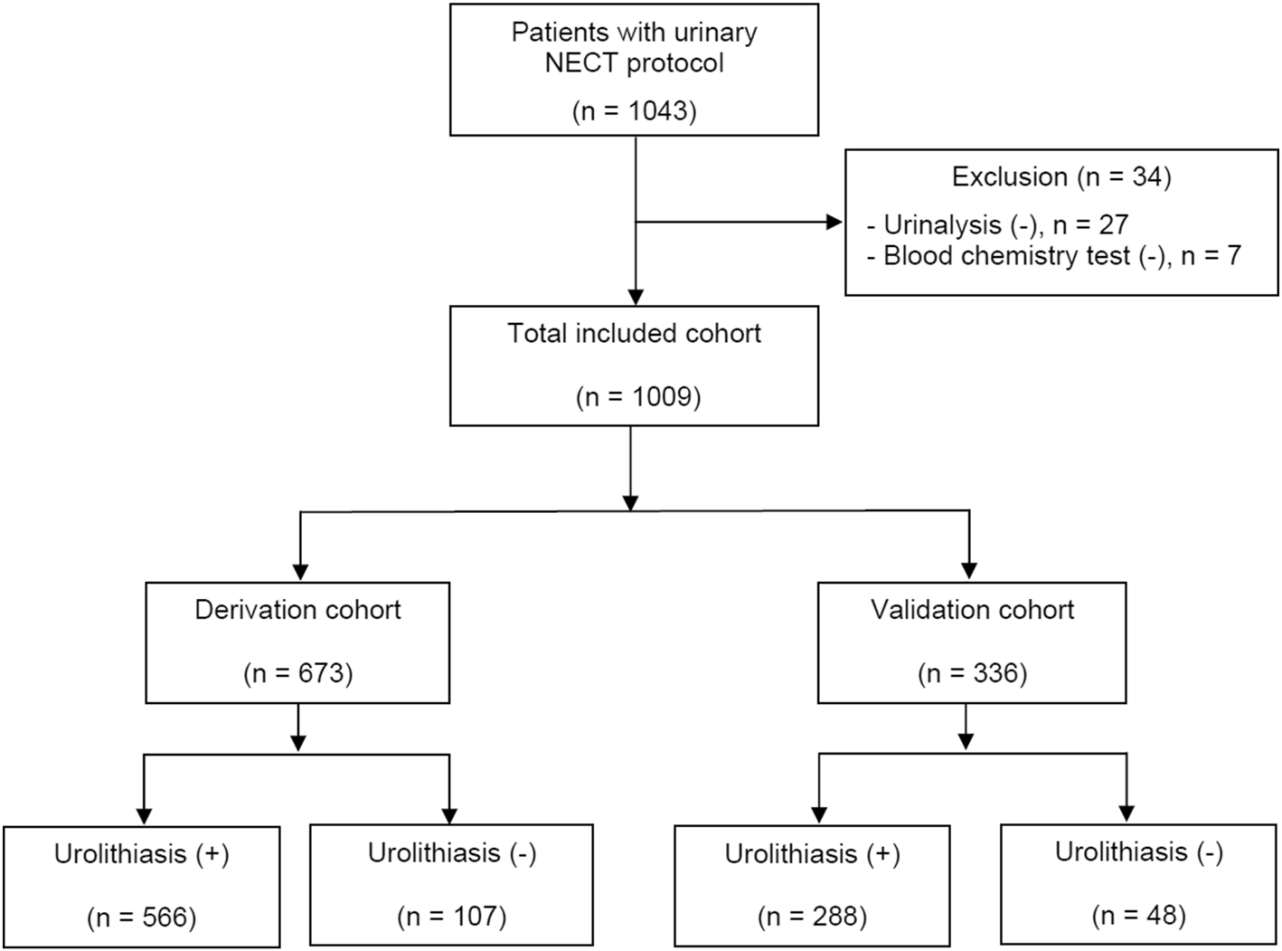
Flowchart for inclusion of patients in the study.

Among the 1043 patients who underwent the urinary NECT protocol with suspected urolithiasis, 34 were excluded due to the lack of urinalysis (n = 27) or blood chemistry results (n = 7) (Fig. 1). The remaining 1009 patients were divided into derivation (n = 673) and validation cohorts (n = 336).

### Derivation cohort

**Figure 2:**
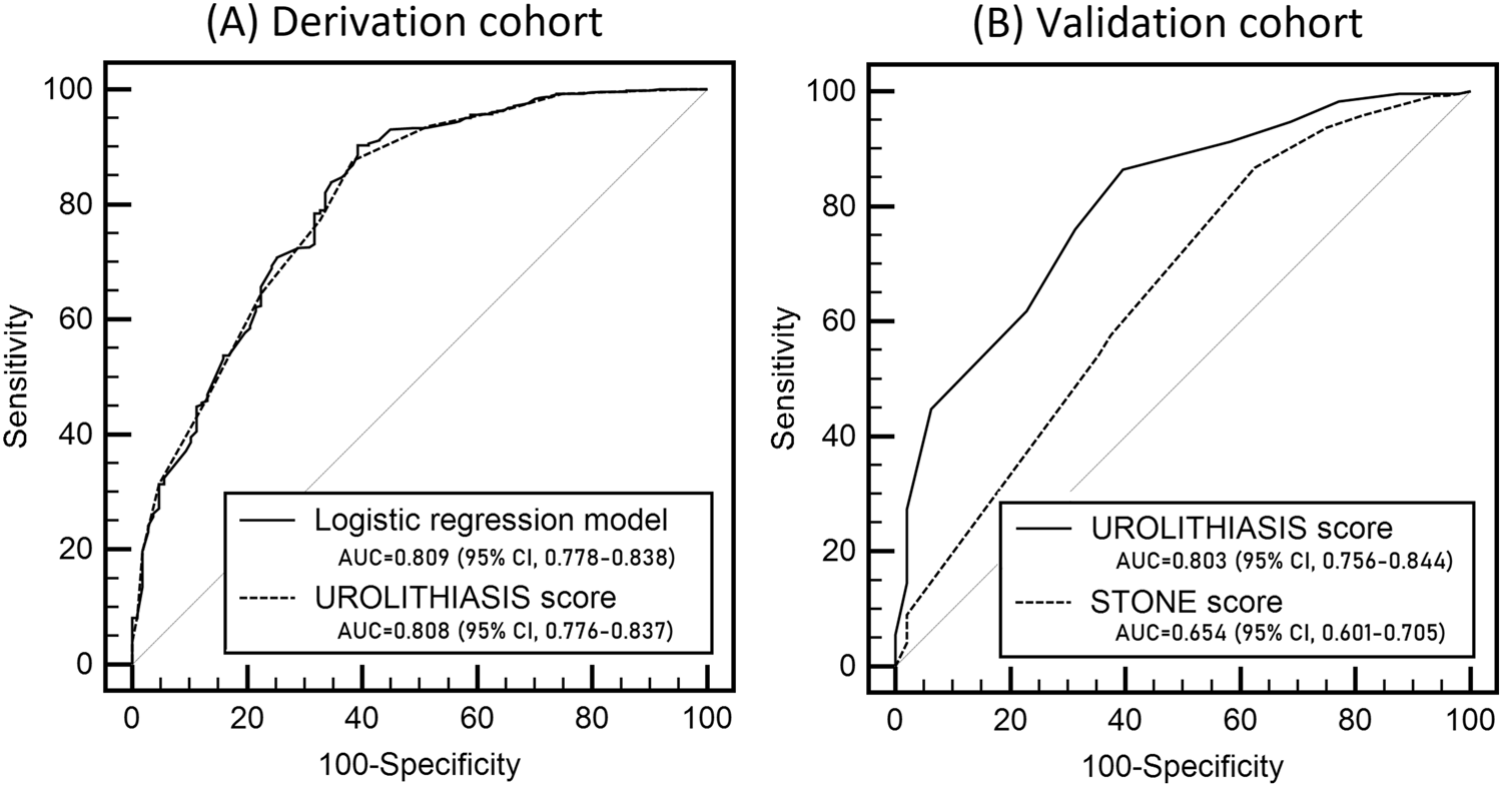
Prognostic performance of the logistic regression model and UROLITHIASIS score in the derivation cohort (**A**) and of the UROLITHIASIS score and the STONE score in the validation cohort (**B**).

In the derivation cohort, 566 patients (84.1%) were diagnosed with urolithiasis and were categorized into the urolithiasis group, and 107 patients (15.9%) were categorized into the nonurolithiasis group. Table 1 shows that several factors were significantly different between the two groups. According to the univariate analyses, sex, age, history of urolithiasis, pain duration, pain score on to the visual analog scale (VAS), nausea and vomiting, costovertebral angle (CVA) tenderness, serum blood urea nitrogen (BUN), creatinine, serum C-reactive protein (CRP), pyuria, hematuria and crystalluria were associated with the presence of urolithiasis. The multivariate analysis yielded 9 factors that were independent predictors of urolithiasis: age > 35 years, history of urolithiasis, pain duration < 8 h, nausea/vomiting, CVA tenderness, red blood cells (RBCs) ≥ 10/high-power field (HPF), no leukocyte ≤  + , any crystalluria and creatinine ≥ 0.92 mg/dL (Table 2). This final model showed good discrimination with an area under the curve (AUC) of 0.809 (95% confidence interval [CI], 0.778–0.838) (Fig. 2A).

**Table 1:** Characteristics of derivation cohort.

	Urolithiasis group (n = 566)	Nonurolithiasis group (n = 107)	*P*
Male	387 (68.4)	57 (53.3)	0.002
Age, years	47.8 ± 13.6	44.1 ± 17.0	0.034
Hypertension	94 (16.6)	11 (10.3)	0.098
Diabetes mellitus	33 (5.8)	8 (7.5)	0.514
History of urolithiasis	171 (30.2)	12 (11.2)	< 0.001
Body temperature, °C	36.5 ± 0.4	36.5 ± 0.5	0.081
Pain duration, h	2.4 (1.1–6.3)	4.7 (1.0–18.5)	0.010
Pain scales*	5.1 ± 2.2	4.3 ± 2.4	0.002
Nausea	124 (21.9)	12 (11.2)	0.012
Vomiting	83 (14.7)	6 (5.6)	0.011
Costovertebral angle tenderness	344 (60.8)	54 (50.5)	0.047
C-reactive protein, mg/dL	0.08 (0.04–0.19)	0.10 (0.03–0.35)	0.067
Blood urea nitrogen, mg/dL	16.7 ± 5.4	15.6 ± 5.5	0.053
Creatinine, mg/dL	1.0 ± 0.4	0.9 ± 0.3	< 0.001
Leukocyte esterase			< 0.001
0	344 (60.8)	68 (63.6)	
±	162 (28.6)	17 (15.9)	
+	43 (7.6)	8 (7.5)	
++	10 (1.8)	11 (10.3)	
+++	7 (1.2)	3 (2.8)	
Occult blood			< 0.001
0	11 (1.9)	24 (22.4)	
±	16 (2.8)	8 (7.5)	
+	40 (7.1)	4 (3.7)	
++	75 (13.3)	8 (7.5)	
+++	424 (74.9)	63 (58.9)	
Nitrite	5 (0.9)	3 (2.8)	0.093
Specific Gravity	1.02 ± 0.01	1.02 ± 0.01	0.449
Red blood cell			< 0.001
0	7 (1.2)	13 (12.1)	
1–3	25 (4.4)	22 (20.6)	
4–9	43 (7.6)	11 (10.3)	
10–19	55 (9.7)	7 (6.5)	
20–29	43 (7.6)	5 (4.7)	
30–49	53 (9.4)	6 (5.6)	
50–99	81 (14.3)	11 (10.3)	
≥ 100	259 (45.8)	32 (29.9)	
White blood cell			0.001
0	103 (18.2)	26 (24.3)	
1–3	280 (49.5)	37 (34.6)	
4–9	131 (23.1)	27 (25.2)	
10–19	37 (6.5)	7 (6.5)	
20–29	7 (1.2)	1 (0.9)	
30–49	2 (0.4)	4 (3.7)	
50–99	3 (0.5)	2 (1.9)	
≥ 100	3 (0.5)	3 (2.8)	
Crystals	55 (9.7)	3 (2.8)	0.019
Stone location			NA
Proximal	165 (30.7)	NA	
Mid	74 (13.8)	NA	
Distal	298 (55.5)	NA	
Stone size, mm	4.3 ± 2.2	NA	NA

Data are presented as n (%) for categorical variables and mean ± standard deviation for continuous variables.

*Visual Analog Scale (1–10).

**Table 2:** Independent predictors of urolithiasis and the assigned scores in the derivation cohort.

	β-coefficient	Odd ratio (95% CI)	*P*	Assigned score
Male	− 0.051	0.950 (0.500–1.805)	0.875	
Age > 35 years	0.615	1.850 (1.126–3.038)	0.015	1
History of urolithiasis	1.011	2.749 (1.398–5.408)	0.003	2
Pain duration < 8 h	0.675	1.963 (1.170–3.294)	0.011	1
Only nausea	0.924	2.520 (0.985–6.443)	0.054	2
Nausea with vomiting	1.368	3.926 (1.493–10.325)	0.006	3
Costovertebral angle tenderness	0.520	1.683 (1.041–2.718)	0.034	1
Creatinine ≥ 0.92 mg/dL	1.112	3.040 (1.649–5.604)	< 0.001	2
Red blood cells ≥ 10/high-power field	1.856	6.398 (3.772–10.851)	< 0.001	4
No leukocyte ≤ +	1.588	4.896 (1.939–12.364)	0.001	3
Any crystalluria	1.407	4.084 (1.165–14.317)	0.028	3
Total scores				20

The UROLITHIASIS score was developed based on these independent predictors. The regression coefficients of the 9 predictors were converted to integer points, which are listed in Table 2. The minimum sum of points was 0, and the maximum was 20. The performance of the UROLITHIASIS score was similar to that of the multivariate model (AUC 0.808 [95% CI, 0.776–0.837]) (Fig. 2A).

**Figure 3:**
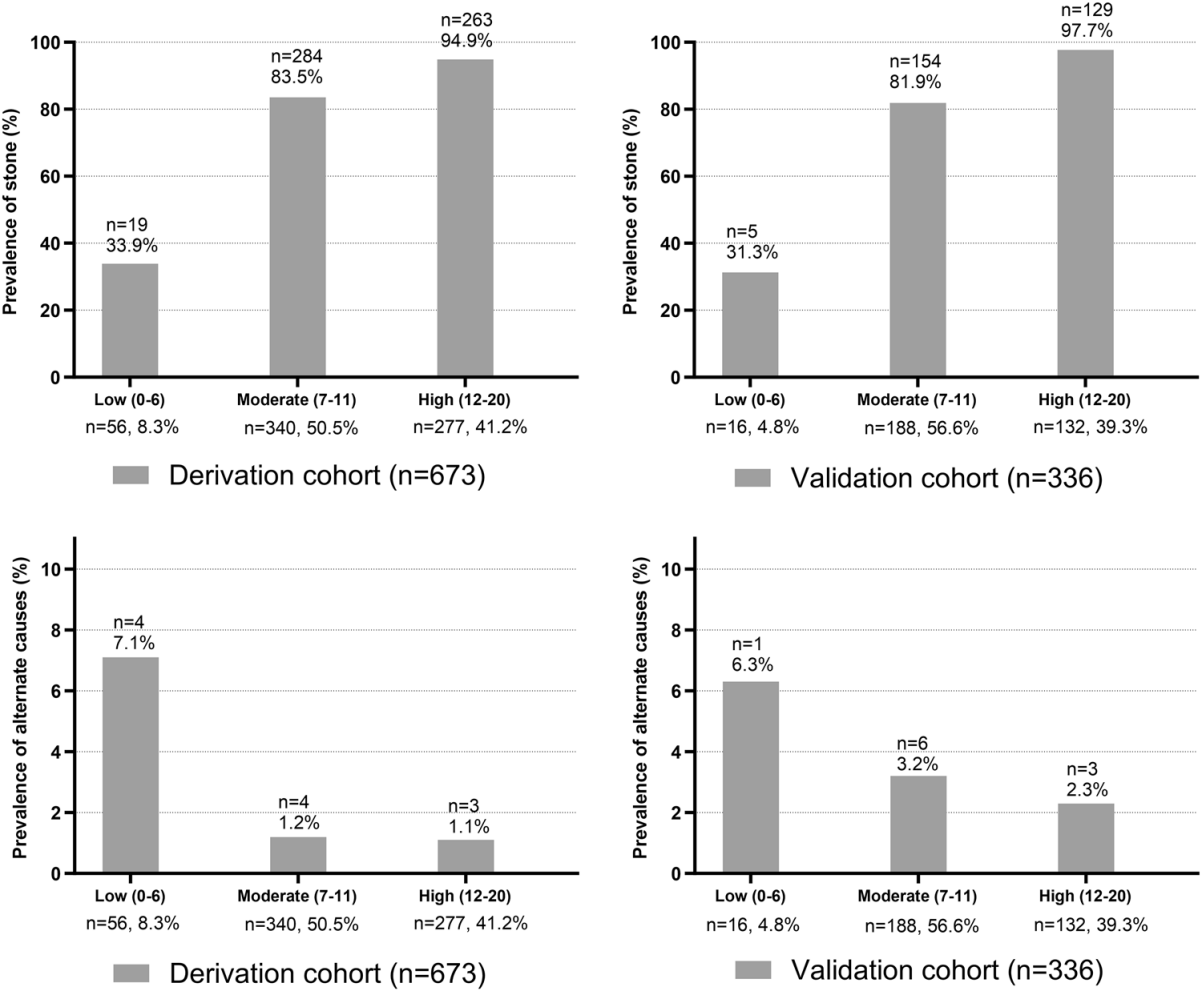
Prevalence of urolithiasis and of important alternate causes by UROLITHIASIS score category in the derivation and validation cohorts.

Figure 3 represents the prevalence of urolithiasis and important alternate causes of symptoms in the 3 risk-stratified groups. A total of 94.9% of the patients in the high-probability (12–20) group were diagnosed with urolithiasis. In the moderate (7–11) and low (0–6) groups, the prevalence of urolithiasis decreased to 83.5 and 33.9%, respectively. On the other hand, as the score decreased from the high- to the moderate- and low-probability groups, the prevalence of important alternate causes increased by 1.1, 1.2, and 7.1%, respectively. The findings of important alternate causes of symptoms are presented in Table 3.

**Table 3:** Important alternate causes of symptoms in the derivation and validation cohorts.

	Derivation cohort	Validation cohort
Pyelonephritis	1	3
Acute renal infarction		1
Pelvic inflammatory disease	1	1
Teratoma	2	
Tubo-ovarian abscess	1	
Ovary cyst rupture	1	
Appendicitis	2	
Diverticulitis	2	
Hemoperitoneum		1
Intra-abdominal abscess		1
Gall bladder cancer		1
Colon cancer		1
Bladder cancer		1
Cholangiocarcinoma	1	

### Validation cohort

Of the 336 patients, 288 were diagnosed with urolithiasis. The AUC of the UROLITHIASIS score was 0.803 (95% CI, 0.756–0.844) (Fig. 2B). The prevalence of urolithiasis and important alternate causes by the three groups was 97.7 and 2.3% in the high-probability group, 81.9 and 3.2% in the moderate-probability group, and 31.3 and 6.3% in the low-probability group, respectively (Fig. 3 and Table 3).

We next investigated whether the STONE score could reliably predict urolithiasis in our validation cohort. The AUC of the STONE score in our cohort was 0.654 (95% CI, 0.601–0.705), which was significantly lower than that of the UROLITHIASIS score (vs. 0.803 [95% CI, 0.756–0.844], *P* < 0.001) (Fig. 2B). According to the STONE score, the prevalence of urolithiasis and important alternate causes was 91.0% and 3.3% in the high-probability group (n = 184), 81.6 and 2.7% in the moderate-probability group (n = 147), and 40.0 and 0.0% in the low-probability group (n = 5), respectively (Table 4).

**Table 4:** Prevalence of urolithiasis and alternative cause by the STONE score category in this external validation cohort and original validation cohort.

Risk categories of the STONE score	Frequency of probability group (%)			Prevalence of stone (%)			Prevalence of alternative cause (%)		
	This validation cohort (n = 336)	Original validation cohort (n = 491)	*P*	This validation cohort (n = 336)	Original validation cohort (n = 491)	*P*	This validation cohort (n = 336)	Original validation cohort (n = 491)	*P*
Low (0–5)	5 (1.5)	76(15.5)	< 0.001	2 (40.0)	7 (9.2)	0.093	0 (0.0)	15 (4.9)*	0.251*
Moderate (6–9)	147 (43.8)	230(46.8)		120 (81.6)	118 (51.3)	< 0.001	4 (2.7)		
High (10–13)	184 (54.8)	185(37.7)		166 (91.0)	164 (88.6)	0.624	6 (3.3)	3 (1.6)	0.307

*Evaluated in the pooled low- and moderate-probability groups.

When comparing our validation cohort with the original STONE score validation cohort, there was a significant difference in the proportion of each probability group (*P* < 0.001) (Table 4). In contrast, the proportions of urolithiasis and alternate causes in each category were not significantly different between two cohorts, except for urolithiasis in the moderate group.

The UROLITHIASIS score did not show a correlation with stone size (r = 0.007, *p* = 0.909) or an association with stone location (Supplementary Tables S1 and S2).

## Discussion

In this retrospective analysis in a large population of patients who were highly suspected of having urolithiasis, a comprehensive clinical prediction score using data easily obtained at the time of ED admission accurately estimated the risk of urolithiasis. Simultaneously, this tool can be inversely associated with the likelihood of an important alternative cause of symptoms. In the validation cohort, the diagnostic performance of the UROLITHIASIS score was significantly higher than that of the STONE score.

In the present study, we developed a new clinical scoring system for urolithiasis that consists of nine independent predictors. Among these, a short duration of pain, a history of urolithiasis, hematuria and nausea/vomiting have also been used in the STONE score or modified STONE score^13,16^. In particular, the presence or absence of hematuria is a point of interest, as its absence may prompt physicians to order more diagnostic CTs to narrow the list of differential diagnoses^19,20^. Hematuria is identified as the most robust predictor of urolithiasis not only clinically but also in scoring systems^21,22^, but different studies define it differently^13,16^. Positive hematuria on the dipstick test is simply a color change due to oxidation of a test-strip reagent, and this does not confirm the presence of RBCs in urine. Various factors can cause false-positive results on a dipstick test^23^. Thus, finding hematuria on the dipstick must be confirmed by microscopic examination. In contrast, if the specific gravity of the urine is very low, microscopy can fail to detect urinary RBCs because they can be lysed with hemoglobin release^23^. Comparing urine dipstick and microscopic examinations to derive our scoring system, we found that the latter had a higher diagnostic value, and the presence of 10 or more RBCs was calculated as a cutoff for urolithiasis, which is a higher value than the positive microhematuria definition according to the American Urological Association (AUA) guidelines (≥ 4 to 9 RBCs/HPF)^24^. A possible explanation for this is that our cohort was selected by the physician gestalt, which can be defined as a physician’s implicit probability estimation based on experience and clinical perception (i.e., presence of microhematuria). This aspect should be taken into account in the interpretation of our results.

Several factors missing from other scoring systems were included in the UROLITHIASIS score. Crystalluria is considered a marker of urine supersaturation, which may be the result of certain metabolic disorders and disproportional urinary inhibitors and promoters, which may ultimately lead to the formation of urolithiasis^25^. From routine serum biochemistry results, we found a significant association between the serum creatinine levels and urolithiasis. Elevated serum levels of creatinine may indicate a high degree of urinary tract obstruction by urolithiasis. Biomarkers for infection may help physicians exclude urinary tract infections (UTIs) that commonly mimic urolithiasis. While Kim et al. revealed the usefulness of serum CRP^16^, leukocyte esterase was included in our final scoring model. Reports regarding urinary markers for UTI are conflicting, some studies showing comparable performance between urine dipstick and microscopy and other studies showing comparable but better sensitivity and diagnostic performance of leukocyte esterase^26,27^. The prevalence of urolithiasis increases with age, and CVA tenderness is present in 25–52% of patients^21,28^. Finally, our comprehensive scoring system showed excellent overall performance in both the derivation and validation cohorts.

Our finding that the overall performance of the STONE score was fair (AUC 0.654) is inconsistent with previous studies^14,16–18,29–32^. The majority of analyses have showed that the STONE score can predict urolithiasis well: its AUCs have ranged from 0.75 to 0.92, and the prevalence of urolithiasis in the high-risk group ranges from 72.7 to 98.7%. However, in contrast to the original study that included only patients undergoing NECT for suspected uncomplicated urolithiasis, several researchers analyzed patients with flank pain, and various CT protocols to evaluate the abdomen were used regardless of contrast use^16–18^. In this study, we validated the STONE score by evaluating patients to undergo urinary NECT scan imaging as a clinical pathway for a patient with suspected urolithiasis. This group likely represents those patients for whom attending emergency physicians (EPs) have greater certainty about the diagnosis of urolithiasis compared with those who received enhanced abdominal CT imaging and were excluded from this analysis. Therefore, patients deemed to have a very high probability of urolithiasis may have been more likely to be included, which was partially confirmed by our rate of urolithiasis and hematuria. The overall prevalence of urolithiasis in the study population was approximately 85%, which is higher than those of the original study (49.5%) and other validation studies. Furthermore, only 67 (10%) of the patients did not present with hematuria according to the AUA guidelines^24^.

According to the UROLITHIASIS score, the distribution of urolithiasis probabilities in our validation cohort was 39.3, 56.6, and 4.8% in the high-, moderate-, and low-probability groups, in which the high- and low-probability groups had corresponding prevalences of urolithiasis and alternate causes, respectively. When we applied the STONE score to our validation cohort, more than half of the patients were categorized in the high-probability group, and 91.0% of them had urolithiasis. Interestingly, the proportions of urolithiasis in the high- and low-probability group were similar to those of the original validation cohort^13^. However, the STONE score indicated that additional CT imaging was necessary for only 1.5% of the patients (low-probability group), of whom 40% had urolithiasis. We believe that our comprehensive score would be more informative for EPs in deciding upon NECT imaging for patients with highly suspected urolithiasis.

There are several limitations to this study. First, it was from a single hospital, and it was a retrospectively designed study. During the study period, suspected urolithiasis patients were managed through the same clinical pathway, but patients were not included if they did not have a CT scan. Additionally, patients with suspected abdominal disease who underwent contrast-enhanced CT were not enrolled in this study, which could lead to selection bias. However, considering the purpose of this score, we believe that the development of the score by evaluating selected patients is more suitable for the future application of this score. Second, increasing the number of clinical factors within a predictive scoring model could lead to a reduction in its practicality even as its predictive power is enhanced. While the STONE score comprises five factors, which are relatively easily to ascertain, the UROLITHIASIS score encompasses nine independent predictors. Furthermore, it encompasses the results of laboratory tests and urinalysis, including parameters such as crystalluria. Nonetheless, considering the excellent predictive performance demonstrated by the UROLITHIASIS score, this comprehensive clinical prediction score could provide even more valuable information, especially about emergency patients with a suspected urolithiasis who have already undergone comprehensive blood tests and urinalysis. Third, the UROLITHIASIS score was primarily designed for diagnostic purposes and is not associated with additional information such as stone size and location, so it cannot guide additional therapeutic decisions. Fourth, urolithiasis may differ in prevalence according to race/ethnicity, geographical conditions, lifestyle, and dietary factors^33,34^, and race is one of the factors of the STONE score, but our score did not include these epidemiological variables. Finally, there was a lack of an assessment of the reliability of the predictor variables. Further prospective studies in different settings should validate this risk score to show the generalizability of our results.

In conclusion, we derived and validated a comprehensive urolithiasis prediction score using various factors, including clinical, blood chemistry and urinalysis parameters in patients with suspected urolithiasis. This clinical scoring system accurately predicted the likelihood of urolithiasis and can aid in patient-centered diagnostic imaging decisions. Further external validation in different settings is warranted.

## Methods

### Study design and patients

We performed a retrospective analysis of consecutive patients admitted to a large urban ED from 2014 to 2015. During the study period, our institution had a clinical pathway for patients with suspected urolithiasis, a protocol that included pain management and urinary NECT scanning by an EP and outpatient follow-up after discharge. The use of POCUS for these patients was not recommended. Accordingly, the attending EP or senior emergency medicine resident examined all adult patients and decided to perform urinary NECT when urolithiasis was suspected. If another abdominal disease or complicated urolithiasis was suspected, a CT scan with the intravenous administration of contrast medium was done.

This study included adult patients (> 18 years) who underwent the urinary NECT protocol. The exclusion criteria included patients without urinalysis or blood biochemistry tests.

The study protocol was approved by the Institutional Review Board (IRB) of the Seoul St. Mary’s hospital (KC17RESI0747) and was performed in accordance with the ethical guidelines of the Declaration of Helsinki. Informed consent was waived by IRB of the Seoul St. Mary’s hospital because of the retrospective nature of the study.

### Data collection

We retrieved the records of patients who underwent the urinary NECT protocol from the picture archiving and communication system. One author reviewed the patients’ medical charts, and all CT scan reports were dictated by board-certified radiologists. From the medical records, clinical variables (sex, age, preexisting disease, body temperature, pain duration, pain scale according to VAS, presence or absence of nausea with/without vomiting and the CVA tenderness) and blood chemistry results (CRP, BUN, creatinine) were collected for all included patients. We analyzed both results that were obtained by the dipstick test and those obtained by microscopic inspection. The following dipstick findings were evaluated: specific gravity, nitrite, occult blood, and leukocyte esterase positivity. The urine samples were also evaluated with a high magnification (400×) microscope after centrifugation at 1500 rpm for 5 min and were recorded as follows: RBC and white blood cell counts/HPF and the presence of any crystals.

### Outcome measures

We acquired CT scans on a multidetector computed tomography scanner (SOMATOM Sensation 64, Siemens AG, Forchheim, Germany), which scanned all patients with a 2-mm slice from the upper pole of the kidneys to the lower edge of the bladder without intravenous contrast medium. Urolithiasis was defined as the presence of one or more attenuation foci in any location from the collecting system to the bladder that was consistent with the patient’s subjective symptoms. Patients having an indirect stone sign (without a visualized stone in the urinary tract but with any acute obstructive signs on urinary NECT) were also considered to have urolithiasis. Because renal pelvic stones can be detected as incidental findings^21^, renal stones without obstructive signs that were located on the opposite side from the pain were considered incidental, and these patients were classified into the nonurolithiasis group. We also documented important alternative causes of the patients’ symptoms from the dictated reports.

### Statistical analysis

All data are presented as the number (percentage) of patients in each group for categorical variables and the mean ± standard deviation or median with interquartile range for continuous variables. To compare the variables between the urolithiasis and nonurolithiasis groups, we used the chi-square test for categorical variables and Student’s t-test for continuous variables.

The enrolled patients were randomly divided into a derivation cohort consisting of 2/3 of the patients and an internal validation cohort with the remaining 1/3 of patients. To derive the risk score models for urolithiasis, we performed univariate analyses between the two groups, and variables with a possible predictive value (*p* < 0.05) in the univariate analyses were included in the multivariate logistic regression analysis. The optimum cutoffs for continuous variables having statistical significance were calculated using Youden’s J statistic. Finally, to simplify this score, the β coefficients of the significant variables were converted to integer points. The UROLITHIASIS score represented the sum of the weighted scores.

To assess the model performance, the developed score was evaluated using receiver operating characteristic (ROC) curve analysis. According to the UROLITHIASIS score, patients were stratified into low-, moderate-, and high-probability groups, and the prevalence of urolithiasis and important alternative diagnoses were determined for each group. Finally, using the validation cohort, we validated the UROLITHIASIS score and the STONE score. Pairwise AUC comparisons were performed between the prediction models. The association between the UROLITHIASIS score and stone size was assessed as Pearson’s correlation coefficient.

All statistical analyses were performed using SPSS version 24 (IBM, SPSS Inc., Chicago, IL, USA). A *p* value < 0.05 was regarded as statistically significant.

### Supplementary Information

Supplementary Information.

## Data Availability

The datasets used and/or analyzed during the current study are available from the corresponding author on reasonable request.
