# Supplementary material for: Comprehensive prediction of urolithiasis based on clinical factors, blood chemistry and urinalysis: UROLITHIASIS score
Source: Sci Rep. 2023 Sep 9;13:14885. doi: 10.1038/s41598-023-42208-9 (PMC10492849; doi:10.1038/s41598-023-42208-9)
Supplement: Supplementary file 1 — Supplementary Information. [file 41598_2023_42208_MOESM1_ESM.docx]

**Supplementary materials**

**Supplementary Table S1**. Prevalence of urolithiasis and of alternative causes according to side of pain and risk-stratified group in the validation cohort (n=336).

|  | Right side (n=171) | | | Left side (n=165) | | |
| --- | --- | --- | --- | --- | --- | --- |
|  | Low-probability (n=6) | Moderate-probability (n=104) | High-probability (n=61) | Low-probability (n=10) | Moderate-probability (n=84) | High-probability (n=71) |
| Urolithiasis | 2 (33.3%) | 79 (76.0%) | 59 (96.7%) | 3 (30.0%) | 75 (89.3%) | 70 (98.6%) |
| Alternate cause | 0 (0.0%) | 3 (2.9%) | 2 (3.3%) | 1 (10.0%) | 3 (3.6%) | 1 (1.4%) |

**Supplementary Table S2**. Differences in the UROLITHIASIS score according to stone location in the validation cohort (n=336).

|  | Proximal (n=95) | Mid (n=29) | Distal (n=145) | P |
| --- | --- | --- | --- | --- |
| UROLITHIASIS score | 11.3 ± 2.2 | 11.5 ± 2.1 | 11.0 ± 2.6 | 0.517 |
